# Amelogenin Supramolecular Assembly in Nanospheres Defined by a Complex Helix-Coil-PPII Helix 3D-Structure

**DOI:** 10.1371/journal.pone.0024952

**Published:** 2011-10-03

**Authors:** Xu Zhang, Benjamin E. Ramirez, Xiubei Liao, Thomas G. H. Diekwisch

**Affiliations:** 1 Brodie Laboratory for Craniofacial Genetics, College of Dentistry, University of Illinois at Chicago, Chicago, Illinois, United States of America; 2 Department of Biochemistry and Molecular Genetics, College of Medicine, University of Illinois at Chicago, Chicago, Illinois, United States of America; University of Crete, Greece

## Abstract

Tooth enamel, the hardest material in the human body, is formed within a self-assembled matrix consisting mostly of amelogenin proteins. Here we have determined the complete mouse amelogenin structure under physiological conditions and defined interactions between individual domains. NMR spectroscopy revealed four major amelogenin structural motifs, including an N-terminal assembly of four α-helical segments (S9-V19, T21-P33, Y39-W45, V53-Q56), an elongated random coil region interrupted by two 3_10_ helices (∼P60-Q117), an extended proline-rich PPII-helical region (P118-L165), and a charged hydrophilic C-terminus (L165-D180). HSQC experiments demonstrated ipsilateral interactions between terminal domains of individual amelogenin molecules, i.e. N-terminal interactions with corresponding N-termini and C-terminal interactions with corresponding C-termini, while the central random coil domain did not engage in interactions. Our HSQC spectra of the full-length amelogenin central domain region completely overlapped with spectra of the monomeric Amel-M fragment, suggesting that the central amelogenin coil region did not involve in assembly, even in assembled nanospheres. This finding was confirmed by analytical ultracentrifugation experiments. We conclude that under conditions resembling those found in the developing enamel protein matrix, amelogenin molecules form complex 3D-structures with N-terminal α-helix-like segments and C-terminal PPII-helices, which self-assemble through ipsilateral interactions at the N-terminus of the molecule.

## Introduction

Amelogenin, the principal protein of the developing enamel matrix, plays a pivotal role in enamel formation and is involved in several Amelogenesis Imperfecta (AI) disease phenotypes [1], [2]. During enamel formation, amelogenin self-assembles into supramolecular structures (nanospheres), which affect enamel crystal size and habit [3]. While it is well established that amelogenin is important for enamel crystal growth, the mechanisms by which amelogenin controls enamel crystal growth remain to be discovered. Determination of the amelogenin structure at the atomic level would be an important step toward understanding how individual amelogenin molecules assemble into supramolecular structures and how these structures contribute to enamel crystal growth.

Difficulties in obtaining protein crystals suitable for X-ray crystallography have prompted a series of studies using circular dichroism (CD), NMR, Raman spectroscopy, and molecular modeling [4]. Earlier CD, FTIR, and Raman spectroscopy experiments suggested mixed β-sheet/β-turn/helix and random coil structures [5]–[8] with extended β-spiral/poly-L-proline type II (PPII) helical structures in the midsection of amelogenin [4]. Recently we have shown that the amelogenin PPII helical region affects nanosphere size and crystal growth [9]. The importance of the amelogenin N-terminus for amelogenin self-assembly has been confirmed by yeast-two-hybrid studies and biochemical analyses of the two serine residues in positions 16 and 25 [10], [11]. NMR studies also suggest that amelogenin assembly is a step-wise process beginning with the N-terminus [12]. Based on solid state NMR data, the amelogenin carboxy-terminal domain appears to be oriented next to the hydroxyapatite crystal surface [13]. Loss of the carboxy-terminus as it occurs during amelogenin proteolytic processing has been associated with a reduced affinity to hydroxyapatite and a reduction in the ability to inhibit crystal growth [14], [15]. Recent crystal growth studies suggest that the carboxy-terminus is important for the alignment of crystals into parallel arrays while the remainder of the molecule plays a role in the inhibition of crystal growth [16].

Two earlier studies have provided important amelogenin structural data, albeit under denaturing or highly acidic conditions. Amelogenin chemical shift assignments without structural predications have been performed under denaturing condition (0.02% HAc) and resulted in the identification of amelogenin candidate regions involved in nanosphere assembly [12]. Another study conducted at pH 3.8 has emphasized intrinsically disordered properties of porcine amelogenin and performed shift comparisons without documenting NMR assignment data [17]. Chemical shift assignment and 3D structure in non-denaturing condition have not been successfully performed, mainly because of the formation of assemblies in full-length amelogenin preparations and because of the extended flexible region in the center of the amelogenin molecule, which leads to severe spectral overlap.

Earlier amelogenin NMR studies focused on highly acidic pH environments (pH 3.8) to circumvent the self-assembly at physiological pH values, which is often detrimental to structure studies. Here we have chosen pH 5.5 as the lowest pH value reported in the physiological enamel matrix [18], [19] and we have focused our structure determination on a large amelogenin center fragment (AA34-154) which yielded identical HSQC spectra at pH 5.5 and pH 7 and was not involved in self-assembly. Structural calculations obtained from the center fragment were then expanded toward the N- and C-terminus using two additional fragments (Amel-N: AA1-92, Amel-C: AA86-180) which were designed to ensure maximum overlap with the center fragment Amel-M and which overlapped with each other.

Combination of individual spectra from these three fragments yielded high similarities between overlapping regions, including chemical shift data, NOE-values, and J-coupling values, prompting us to assemble a complete amelogenin structure based on physiological 3D NMR data. In addition, a comparison between the truncated, monomeric Amel-M amelogenin fragment and the fully assembled mouse M180 amelogenin allowed us to map amelogenin domains as they relate to nanosphere assembly under physiological conditions. The Deuterium enriched amelogenin proteins that were used in this study yielded high quality structural assignments, especially of the N-terminal 45 amino acids that form the unique tryrosine-rich amelogenin peptide (TRAP) region. Here we present the first amelogenin structure obtained under physiological conditions found in the enamel matrix and explain key interactions required for amelogenin self-assembly.

## Results

### Amelogenin – a complex molecule with pH-dependent assembly properties

**Figure 1:**
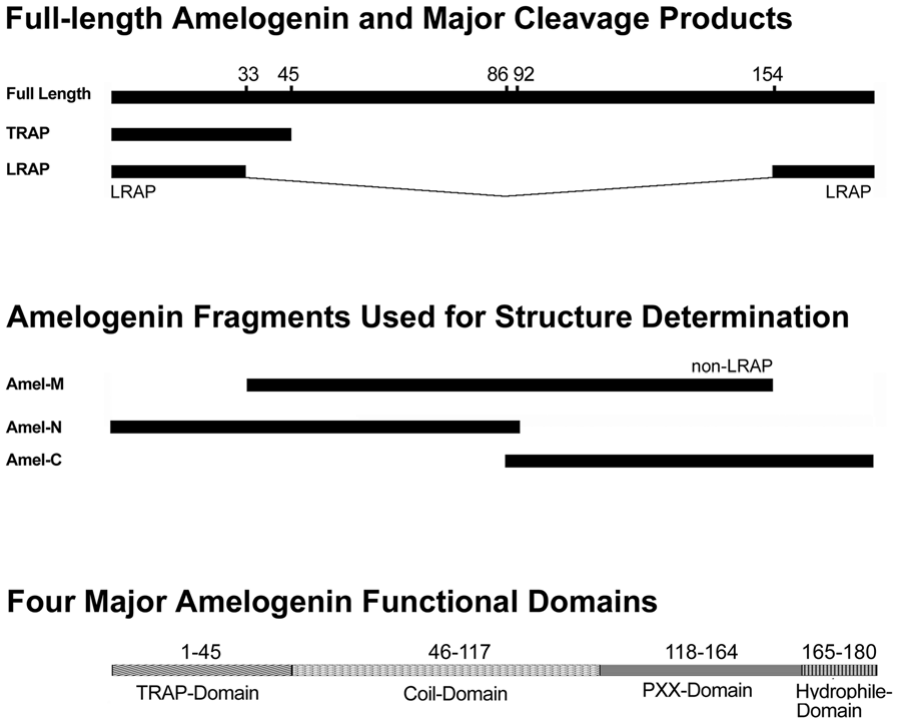
Functional domains and cleavage products of the tooth enamel protein amelogenin. Besides the 180 amino acid full length amelogenin, the developing enamel matrix contains two major fragments, the tyrosine-rich amelogenin peptide (TRAP, AA1–45) and the leucine-rich amelogenin peptide (LRAP, AA1–33 & 155–180). For our amelogenin structure determination using 3D-NMR, three fragments were generated: Amel-M (AA1–92), Amel-N (AA34–154, non-LRAP), and Amel-C (AA86–180). Based on our structural data, we now distinguish between four major amelogenin domains, (i) the TRAP domain (AA1–45), (ii) the coil domain (AA46–125), (iii) the PXX repeat domain (AA126–164), and the hydrophilic C-terminal domain (AA165–180).

The full-length amelogenin is a complex molecule consisting of approximately four major functional domains: (i) the N-terminal, tyrosine-rich amelogenin polypeptide TRAP (AA1-45), (ii) a central, histidine-rich region (AA46-125), (iii) the polyproline tripeptide repeat region (AA126-164), and (iv) the hydrophilic and charged C-terminus (AA165-180) (Fig. 1). Amelogenin structural information has been difficult to obtain due to the pH dependent assembly behavior of amelogenin [4]. Initial amelogenin solution NMR studies yielded structural information from polyproline repeat peptides based on the proline-rich repeat region of the protein (∼AA 71–169) and suggesting that the polyproline central domain was amenable to structural studies. Preliminary studies also suggested difficulties with obtaining structural information from the hydrophobic N-terminus (AA 1–33) at pH 7.

### Three-fragment approach facilitates amelogenin NMR structure determination

In order to facilitate the generation of readable NMR spectra at near-physiological pH (pH 5.5), and overcome severe spectral overlap of full length amelogenin, we designed a 121 amino acid fragment (Amel-M, AA 34–154) that included the entire length of the amelogenin central portion excluding the leucine-rich amelogenin peptide (LRAP) flanking portion and excluded the hydrophobic N-terminus (non-LRAP, Fig. 1). Amel-M was of sufficient length to obtain an extended amelogenin spectrum with good resolution and remained monomeric even at pH 7. We then divided the full-length amelogenin into two halves with six amino acids overlap, Amel-N (AA 1–92) and Amel-C (AA 86–180), to ensure fidelity of overlapping spectra. Both Amel-M and Amel-C generated useful spectra at pH 5.5 and at pH 7. The overlapping three-fragment design allowed for dual verification of chemical shifts and thus minimized possible structural effects of N-terminal tags (Table S1).

### Assignment of amelogenin structural data at pH 5.5

**Figure 2:**
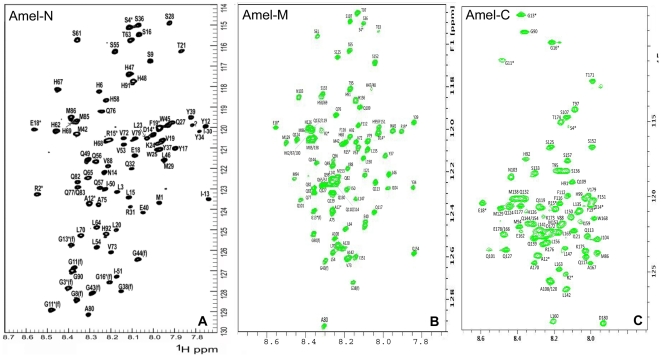
Two-dimensional ^1^H-^15^N HSQC spectra of amelogenin fragments Amel-N, Amel-C, and Amel-M. Two-dimensional HSQC spectroscopy was conducted under identical conditions (pH = 5.5) for all three amelogenin fragments, Amel-N, Amel-M, and Amel-C. Individual peaks were labeled with residue names and numbers, either directly or with arrows. His-tag residue peaks were marked with an asterisk. Aliased or folded peaks were labeled with an (f). Y26 (7.60, 120.22) was not shown in the figure. ^1^H–^15^N HSQC spectra of individual fragments are shown as follows Amel-N (A), Amel-C (B), Amel-M (C).

For chemical shift assignment, double-labeled Amel-N, Amel-M and Amel-C (^15^N and ^13^C, and ^2^H and ^15^N) fragments were generated in *E. coli*. For structural determination use, all NMR spectra (Amel-N, Amel-M, and Amel-C) were collected at pH 5.5. Amel-M spectra were identical at pH 5.5 and pH 7. The backbone and side chain chemical shift assignments were obtained from a combination of 2D and 3D experiments. Figure 2 contains HSQC spectra and corresponding backbone assignments for Amel-N (Fig.2A), Amel-M (Fig.2B) and Amel-C (Fig.2C). For the backbone HN group assignment, all amino acid residues were assigned including 8 amino acid residues that were not separated well in all three fragments, listed as Q77/Q83, H99/F151, Q110/Q114 and Q119/H126. For Cα assignment, 169 out of 180 amino acid residues were assigned and for Hα assignment, 174 out of 180 amino acid residues were assigned. The HSQC spectrum of the N-terminal amino acid residues was better dispersed than that of the C-terminal amino acid residues. Especially the glutamine residues in the amelogenin exon 6-encoded PXQ repeat region tended to overlap, indicating that successive PXQ repeats adopted a repetitive structure with subunits of similar conformation between individual repeats.

### Cα and Hα comparisons, J-coupling, and NOE analyses revealed distinct structural motifs of the full-length amelogenin

**Figure 3:**
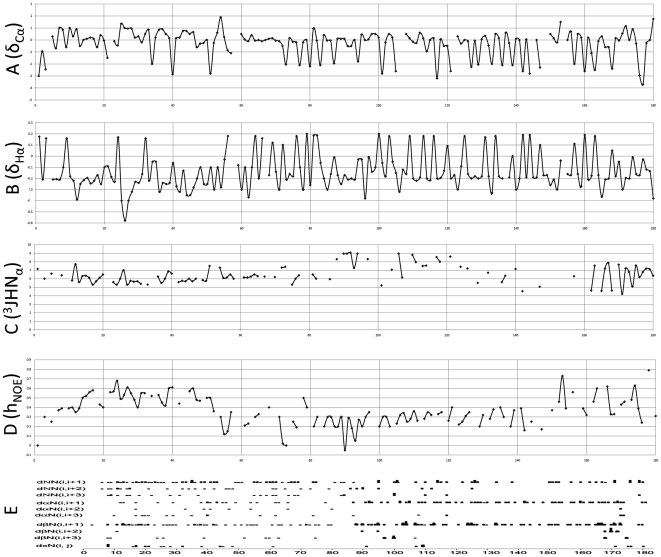
Summary of chemical shift deviations, J-couplings, heteronuclear NOEs and inter-residue NOE signals. (**A** and **B**) δCα and δHα chemical shift differences were obtained by subtracting published amino acid residue average chemical shifts (BMRB Database Statistics) from measured Cα and Hα chemical shifts of amelogenin assigned amino acid residues. **C**, J_HNα(3)_ coupling values of amelogenin amino acid residues. **D**, Summary display of heteronuclear NOE values. The L15-W45 regions featured average NOE values of 0.6 while the central regions of the amelogenin molecule displayed NOEs between 0.5 and −0.2 (0.3 average), suggesting that the L15-W45 region contained a relatively rigid structure while the M1-N14 and L46-D180 regions were more flexible in comparison. **E**, Summary display of Interresidue NOE signals. The interresidue NOEs were classified into dNN, daN and dbN signals and each class included (i, i+1), (i,i+2) and (i, i+3) subcategories. dαN(i,i+1) and dβN(i,i+1) signals were denser between amino acids 100 to 170. Interresidue NOEs equal to or more than four amino acids apart (dxN(i, j)) and interresidue NOE that were three amino acid apart (dNN(i,i+3), dαN(i,i+3), dβN(i,i+3)) were mainly observed at the amelogenin N-terminus.

In order to gain further insights into the amelogenin secondary structure, our previously assigned Cα and Hα chemical shifts were compared with the corresponding amino acid residue average chemical shifts from BMRB Database Statistics (http://www.bmrb.wisc.edu/ref_info/). δCα and δHα values were calculated to represent the Cα and Hα chemical shift differences between amelogenin and BMRB average chemical shifts, respectively (Figs. 3A,B). The graphic representation of δCα and δHα shift differences in Figs. 3A,B illustrate that δCα and δHα were shifted in opposite direction, i.e., wherever δCα was positive, δHα was negative and vice versa. There were altogether 37 δCα values that shifted downfield beyond −1.5 ppm in negative direction. Notably, 34 out of these 37 δCα downfield shifting residues occurred immediately prior to proline residues. The three residues with downfield shifts exceeding −1.5 ppm that did not precede a subsequent proline were Q57 (−2.53 ppm), N103 (−2.78 ppm) and Q113 (−3.03 ppm). Twenty two out of 23 assigned δCα immediately preceding proline residues were smaller than −1.5 ppm. The only exception was residue Q117 (−0.79 ppm), which shifted less than the other pre-proline residue δCα values. The up-field and down-field shifted Cα and Hα plateaus at the N-terminus of amelogenin (residues G8 to W45) were indicative of helix-like structures in this region, except for the interruption at residue P27. In order to verify the presence of helical structures at the N-terminus of amelogenin, J-coupling constants and inter-residual NOE patterns were analyzed. All of the ^3^J_HNα_-coupling values between G8 and W45 were smaller than 7, while no consistent patterns was be detected for the C-terminal sequence, suggestive of helix-like structures for the N-terminus.

Figure 3E illustrates the distribution of inter-residue NOE signals along the amelogenin sequence. Unambiguous NOE connectivities were first determined from NOE-HSQC acquired from deuterium enriched protein. This strategy allowed for optimum resolution of NOE signals due to reduced line-width. More importantly, the selective proton enrichment of amino acids side chains from ^1^H glucose in 95% D_2_O media enhances reliability of NOE assignments. Furthermore due to replacement of protein with deuterium, the degree of spin diffusion is much reduced. The NOE signals between i and i+1 residues for dNN, dαN and dβN were almost uniformly distributed throughout the whole protein, while the NOE signals between i and i+3 residues for dNN, dαN and dβN were mainly identified at the N-terminus. These data further support the concept that the N-terminal amelogenin TRAP region contains a dispersed confirmation consisting of three possibly interacting α-helix-like structures. Together, δCα and δHα chemical shifts, ^3^J_HNα_ values, and heteronuclear NOEs suggested a presence of helical structures at the N-terminal amelogenin TRAP region. Furthermore, heteronuclear NOEs (hNOE) were around 0.6 in the N-terminal region (residues G8-W45) and substantially higher than the hNOEs in the remainder of the molecule (hNOEs approximately 0.3), indicating a higher degree of rigidity at the amelogenin N terminus (AA1-45) (Fig. 3D).

### The amelogenin N-terminal TRAP region (AA1-45) contained several helices

**Figure 4:**
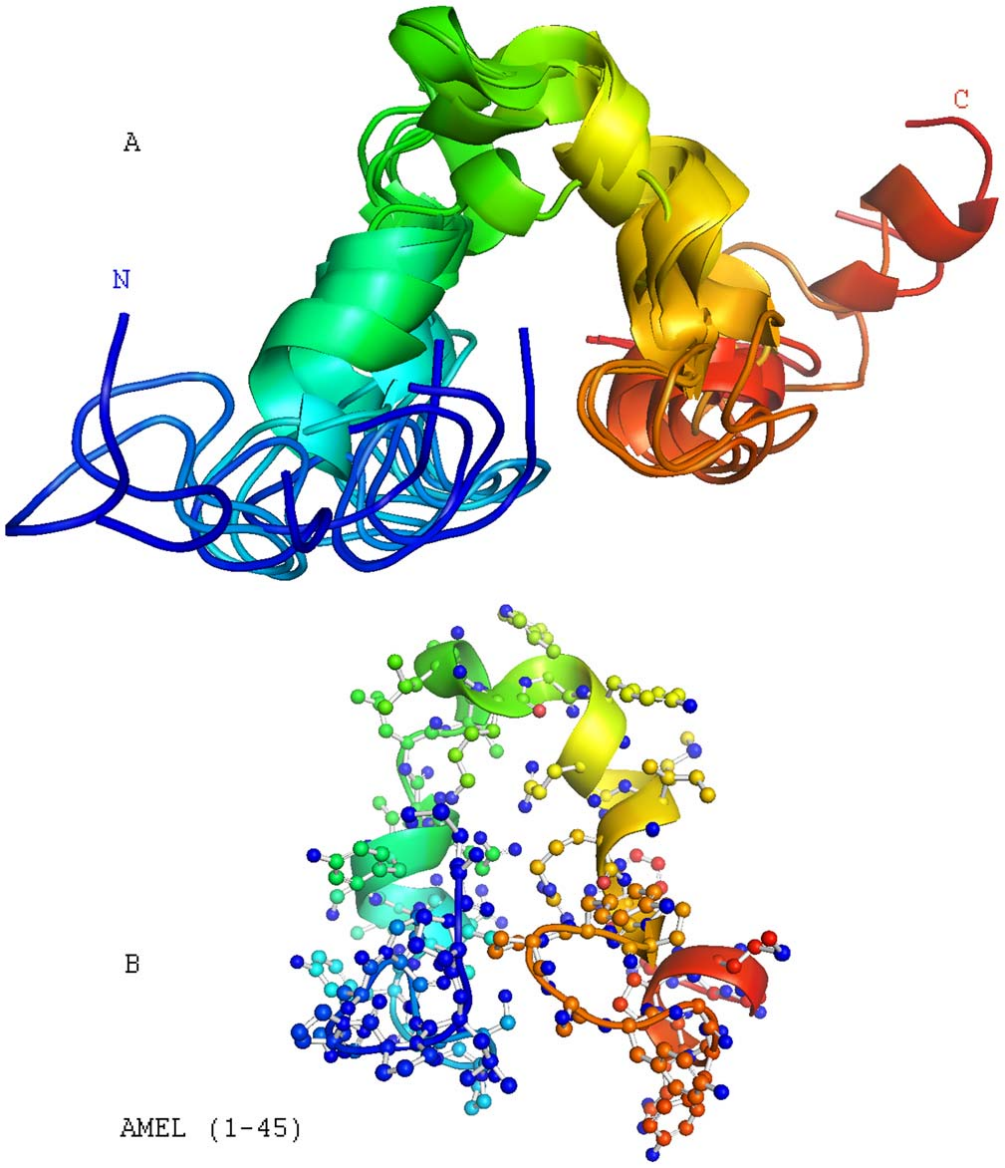
Solution NMR structure of the amelogenin TRAP region (AA1-45). **A**, Structural diagrams based on backbone traces from 6 selected conformers with lowest target functions calculated using the DYANA software program. Superimposed conformers from amelogenin TRAP region (AA1-45). **B**, Ribbon diagram representation of amelogenin TRAP region structure. The two regions (S9-V19) and (K24-I30) adopted α-helix like secondary structure and interacted with each other through Y17 and W25 side-chains. The region between V19-L23 formed a turn.

In order to visualize the solution NMR structure of the TRAP region, backbone traces from 10 conformers with lowest target functions were selected from a family of DYANA structures (Table 1) and subsequently were further refined by molecular dynamics calculations with NMR constraints using SANDER. Finally, the structures were aligned and plotted using MolMol software. Superimposed conformers from the amelogenin TRAP region (AA1-45) illustrate the position of multiple helices (Fig. 4A). A more detailed structural calculation based on the lowest energy conformation highlights two regions with α-helical secondary structure (S9-V19 and K24-I30) modulated through a turn, which interacted among each other through Y17 and W25 side chains, as well as a third helical segment between Y39 and W45 (Fig. 4B). The unique presence of three helical segments at the tyrosine-rich amelogenin N-terminus is indicative of possible N-terminal amelogenin protein-protein interactions in the 3-dimensional enamel matrix assembly.

**Table 1:** Statistics for Mouse Amelogenin Structure Determination.

	mAMEL-N
**(A) NMR-derived constraints**	
Total constraints	485
Sequential (|i-j| = 1)	273
Medium-range (1 < |i - j| < 5)	185
Long-range (|i - j| >4)	26
Hydrogen bonds Dihedral angles (ϕ, ψ)	68
**(B) Residual violations**	
DYANA target function (Å^2^)	
Upper limit	0.85±0.08
Sum (Å)	9.8±1.4
Maximum (Å)	12.7
van der Waals	0.6±0.06
Sum (Å)	20.3±1.8
Maximum (Å)	23.5
**(C) Average r.m.s.d. to mean**	
**structure (Å)**	
Backbone atoms N, Cα, C' (Å)	11.53±2.86
All heavy atoms (Å)	12.25±2.69
**(D) Ramachandran plot (% residues)**	
Residues in most favored regions	62.5%
Residues in additional allowed regions	32.8%
Residues in generous allowed regions	3.1%
Residues in disallowed regions	1.6%
**(E) NMR constrained molecular**	
**dynamics calculation results**	
Total constraints applied in MD	2433 (NOE distances)
Dihedral angles	326 (angles)
RMSD	7.7±1.2 Å
**(D) Ramachandran plot (% residues)**	
Ramachandran plot results	
Residues in most favored regions	87.3%
Residues in additional allowed regions	10.4%
Residues in generous allowed regions	2.3%
Residues in disallowed regions	0%

The table is a summary of input restrains for structure calculation using DYANA and 10 best structure conformations of AMEL-N and followed by NMR constraints calculation using the AMBER package.

### The TRAP-neighboring region (AA46-85) was characterized by turns and coils interrupted by an α-helix and a 3_10_ – helix

**Figure 5:**
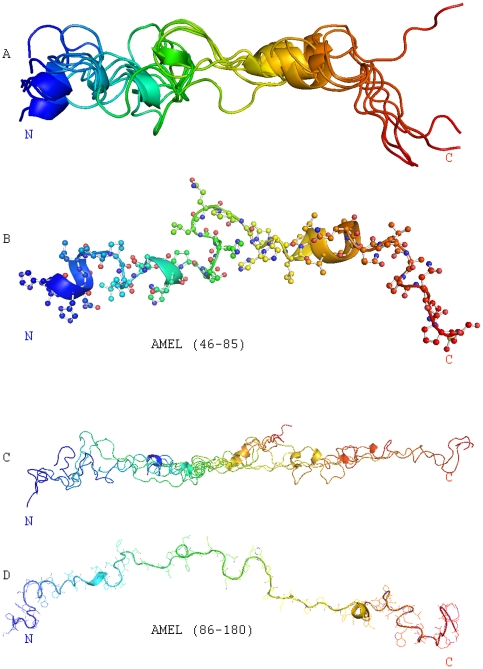
Solution NMR structure of the central and c-terminal amelogenin (AA46-180). A, Alignment of ten selected conformers from the TRAP-neighboring region (AA46-85) with lowest target energies calculated using the DYANA software program. This fragment was characterized by turns and coils interrupted by an α-helix at V53-Q56 and an unusual 3_10_ – helix at P74-Q76. **B**, Backbone ribbon representation and side chain heteroatom representation of one TRAP neighbor region (AA46-85) lowest energy structure. The position of the α-helix and rare 3_10_ – helix are highlighted. **C**, Alignment of ten selected Amel-C conformers (AA86-180) with lowest target energies, once more calculated using the DYANA software program. Note the repetition of fairly similar PXX conformers along the polyproline type II helix region. **D**, Backbone ribbon representation and side chain heteroatom representation of one Amel-C lowest energy structure.

Structural parameters of the TRAP-neighbor region (AA46-85) were visualized as described above. Superimposed conformers from this region (AA46-85) illustrate the position of a α-helix and a 3_10_ – helix amidst multiple turns and coils (Fig. 5A). Detailed structural analsysis based on the lowest energy conformation confirms the α-helix like secondary structure (V53-Q56) and the 3_10_ – helix (P74-Q76) (Fig. 5B). 3_10_–helices are intrinsically less stable than α-helices and might have unique functions in the packing and dissociation of protein assemblies [9], [20], making them ideally suited for crucial roles during conformational changes in the enamel matrix.

### The amelogenin PXX repeat region formed an elongated stretch comprised of three polyproline type II helices

**Figure 6:**
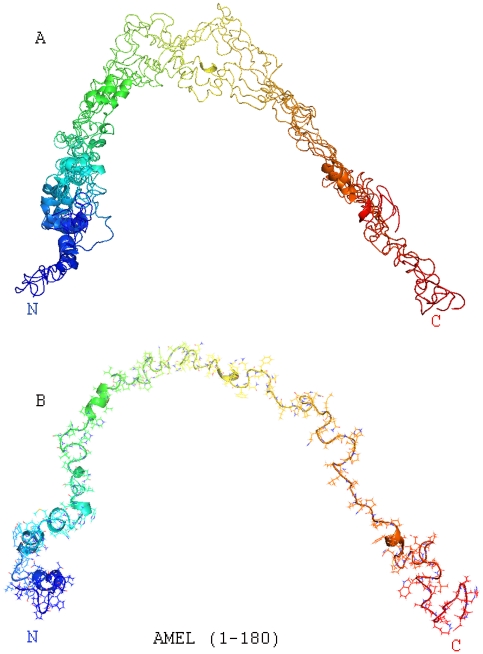
Solution NMR structure of the entire full-length mouse amelogenin (M180). (**A** and **B**), Alignment of 10 lowest energy conformers (**A**) and line structure (**B**) of the full-length mouse amelogenin (M180) based on full-length amelogenin NMR constraints calculated by DYANA. Helices are demarked by cartoons. Note the position of the 3_10_ – helices at P74–Q76.

Heteronuclear NOEs, δCα and δHα chemical shifts, and ^3^J_HNα_-coupling values strongly suggested that the N-terminus of the amelogenin protein adopted disrupted α-helix like structures. However, the absence of consistent downfield or upfield chemical shift stretches and dispersed ^3^J_HNα_-coupling values in the center and at the C-terminus of mouse amelogenin as well as the fairly small heteronuclear NOEs (approximately 0.3), indicated that the C-terminal amelogenin domains (residues 86–180) did not display any traditional protein secondary structure characteristics, with the exception of a second 3_10_ – helix at S107–Q109. The absence of long-range NOEs within the entire Amel-C (Fig. 5C) suggests that Amel-C did not form β-sheets in aqueous environments. The backbone and side chain resonances in the PXX region overlapped substantially. The resonances from GLN residues at the third position were essentially identical with Hγ side chains showing almost identical splitting. These data strongly indicate PXX repeats adopt homologous confirmation in the protein. In contrast to the less-definded organization of the central domain (AA46-125), the extended PXX repeat region (AA126-164) was characterized by a succession of ten fairly similar conformers (Figs. 5D, 6B). These PXX repeats were proposed to form left-handed extended polyproline helices [21], [22]. Due to the extended structure of this type of helix, the number of observable inter-residue NOEs is rather limited and the structural determination is difficult based on NOE connectivities. In order to address difficulties with structure determination based on inter-residue NOEs, dihedral angle constraints deduced from the ideal polyproline helix were introduced into the structural calculation. The ^13^Cα chemical shifts were then predicted from the calculated structure using the program SPARTA [23] and compared with experimentally obtained chemical shift data for the entire amelogenin (Fig. S1). This comparison revealed a good match between predicted and experimental chemical shift data without violation of the limited experimental NOE data. Backbone ribbon and side chain heteroatom representation of one Amel-C lowest structure (Fig. 5D) illustrates a series of three left-handed extended polyproline type II (PPII) helices (PPII-1, Q127-P131; PPII-2, H136-F151; PPII-3, P158-P164). These PPII helices were identified using the following criteria: (i) left-handedness, (ii) 3 amino acid residues per turn, and (iii) 3.1Å per residue advance (9.3Å per turn) (Fig. 5B). The proline rings in the PPII helix at the positions i and i+3 were oriented in the same direction. Individual PPII turns measured 9.48Å (PPII-1), 9.29Å (PPII-2), and 9.42Å (PPII-3).

### The full-length mouse amelogenin structure contained (i) an N-terminal helical domain, (ii) a random coil domain featuring a 3_10_ helix, (iii) a long stretch of unfolded PPII helices, and (iv) a folded C-terminal coil region

In order to calculate the full-length amelogenin structure, mouse amelogenin M180 NMR constraints were collected and used for 3D structure reconstruction aided by the DYANA software package followed by NMR constrain MD calculations using SANDER from the AMBER package. Using this approach, 10 lowest energy conformers were obtained (Fig. 6A, B). A basic analysis identified four regions: (i) an N-terminal domain characterized by a series of four α-helices (AA1- ∼60), (ii) an elongated random coil region interrupted by a 3_10_ helix (∼AA60–117), (iii) a long stretch of unfolded PPII helices (AA118–164), and (iv) a C-terminal coil region (AA165–180)(Fig. 6A,B).

### The mouse amelogenin center region (T63-M138) maintained a monomeric state even in the assembled nanosphere

**Figure 7:**
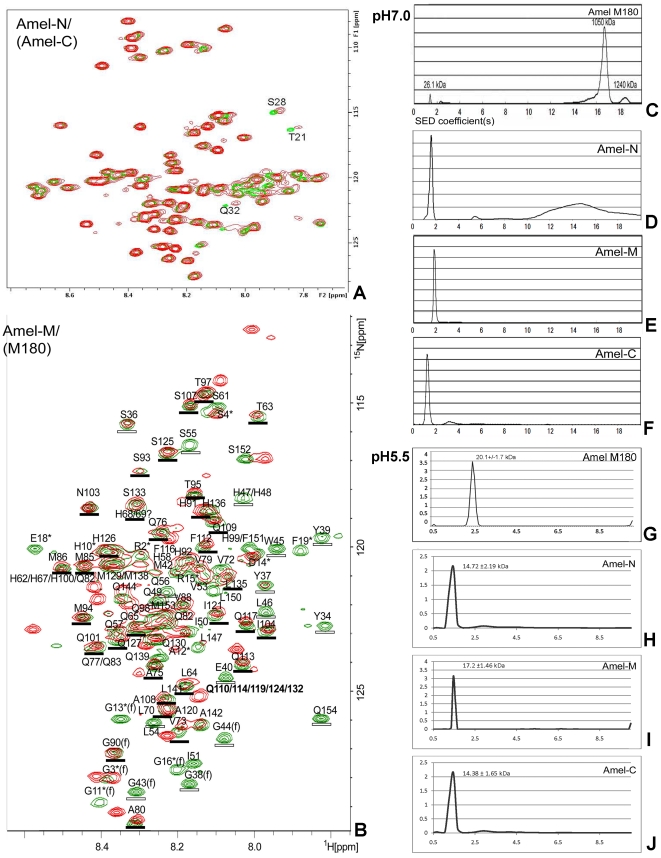
Amelogenin domains and full-length molecule: Interactions and assembly behavior. (**A** and **B**), Interaction between fragments and the full-length amelogenin as revealed by solution NMR. There were only weak interactions between Amel-N and Amel-C at T21, S28, and Q32 (chemical shift) while all others overlapped (**A**). Comparison of Amel-M (green color) and full length mouse amelogenin (red color) two-dimensional ^1^H-^15^N HSQC spectra at neutral pH (**B**). The Amel-M amino acid residue peaks which were absent or dramatic reduced in fAmel spectra were marked with hollow bars, including Y34, S36, Y37, G38, Y39, E40, G43, G44, W45, L46, H47, H48, Q49, I51, V53, L54, S55, Q56, H58. Peaks which were present in both Amel-M and full length spectra were marked with solid bars, including T63, L64, H68, H69, V72, V73, A75, Q76, A80, Q82, Q83, M85, V88, G90, H91, S93, M94, T95, T97, Q101, N103, I104, S107, A108, Q109, F112, Q113, Q117, I121, S125, H126, Q127, M129, Q130, Q132, S133, L135. Weak or lost HSQC peaks between AA34-62 indicate that the N-terminal Amel-M region may be involved in amelogenin nanosphere assembly (**B**). Analytical ultracentrifugation was used to demonstrate the self-assembling properties of Amel-N, Amel-M, Amel-C and of the full-length M180 amelogenin at pH 5.5 and pH 7 (**C**–**J**). **C**, the full-length M180 fragment displayed a monomeric peak at 26.1 kDa and heterogeneous assemblies with sizes between 1050 kDa to 1240 kDa at pH 7.0. (**D**–**F**), two of the three amelogenin fragments employed in this study Amel-M and Amel-C, were mostly monomeric (**E,F**), while Amel-N also featured high molecular weight self-assemblies in addition to a single peak (**D**). (**G–J**), at pH 5.5, all three fragments Amel-N, Amel-M and Amel-C as well as the full-length M180 revealed singular peaks and no higher molecular weight assemblies, suggesting monomeric distribution of these fragments and of the full-length amelogenin at pH 5.5.

Amelogenins interact with each other to form nanospheres, which play important roles during enamel crystal formation [3]. Recent studies analyzing amelogenin nanosphere self-assembly in acidic condition (2% acetic acid, pH 3.0) suggested that the amelogenin N-terminus is important for the interaction between individual amelogenin molecules [12]. Here we have used aqueous conditions to compare HSQC spectra between full length amelogenin and the Amel-M fragment (Fig. 7B), allowing for a comparison of supramolecular assembly properties at near physiological pH. When comparing full-length mouse amelogenin and Amel-M HSQC spectra, there was a complete match between all well-resolved peaks (about 40) in the region between residues T63 to M138. When superimposed, differences between T63-M138 full-length and Amel-M spectra were smaller than 0.2 ppm (^15^N chemical shifts), and smaller than 0.02 ppm (^1^H chemical shifts), suggesting high structural homologies between the T63-M138 stretches of full-length Amel-M amelogenins (Fig. 7B). Together, these data confirm the suitability of the Amel-M spectrum as a strategy to improve NMR structure recognition for the full-length amelogenin structure.

### While the Y34-T63 stretch of Amel-M was involved in nanosphere assembly, the adjacent fragment T63-M138 remained monomeric even in assembled nanospheres

Comparing full-length with Amel-M amelogenin resulted in a significant degree of structural homology of the T63-M138 domain between assembled and non-assembled amelogenins indicated that this region was un-affected by the 3D assembly of amelogenin nanospheres (Fig. 7B). In comparison, peaks identified in the N-terminal Amel-M helical region Y34 to T63 were absent or greatly reduced in the full length amelogenin spectrum, indicating that this region was involved in full-length amelogenin nanosphere assembly (Fig. 7B). Together, these experiments define the region between T63 to M138 as a stretch that remains monomeric even in assembled nanospheres. Other domains such as Q139-Q154 also lacked NMR signals. These are likely to play are role during conformational changes related to self-assembly since according to AUC the Amel-M fragment overall did not self-assemble (Fig. 7H).

### N- and C-terminus of M180 did not interact with each other

In order to determine a potential relationship between the amelogenin N- and C-terminus, the amelogenin C-terminal fragment Amel-C (AA86-180) was added to the ^15^N-labelled N-terminal rM180 fragment Amel-N (AA1-92) and HSQC spectra were recorded before and after addition of the C-terminal fragment. HSQC ^1^H-^15^N spectroscopy of the N-terminal fragment with or without addition of a C-terminal amelogenin fragment resulted in almost identical and overlapping spectra (Fig. 7A), indicating that the N-terminus and the C-terminus of rM180 did not interact with each other in solution.

### Selfassembly and solubility behavior of amelogenin fragments depend on pH and temperature

Previous studies have reported on the intriguing pH- and temperature- dependency of amelogenin self-assembly [24]–[26]. During the course of our studies we found that while the full-length amelogenin was soluble at pH 7.0, the N-terminal fragment Amel-N was only soluble at pH 6.0 and below. We were thus interested in asking the question how individual amelogenin fragments contribute to amelogenin solubility and self-assembly. To address this question, we once more used our Amel-N, Amel-M, and Amel-C fragments and determined their self-assembly properties using analytical ultracentrifugation and NMR spectroscopy. NMR spectra revealed that Amel-N formed supramolecular assemblies at pH 7.0 (not shown) while it became monomeric only at pH 5.5 (Fig. 2A), suggesting that the Amel-N fragment maintained self-assembly properties for nanosphere formation at neutral pH, but lost its solubility.

In contrast, Amel-M was soluble and monomeric under neutral and slightly acid conditions, as revealed by similar sedimentation graphs (Fig. 7E and H) and NMR spectra at pH 5.5 and pH 7 (Fig. 2B). This finding is supported by the monomeric state of a long stretch in Amel-M (T63-M138) even in the assembled amelogenin nanosphere (Fig. 7B). Finally, the Amel-C fragment was soluble but formed supramolecular assemblies at neutral pH (Fig. 7B). These data identify the central region of the amelogenin molecule (T63-M138) as the most soluble portion of the molecule and the one least likely to assemble, the N-terminal (Amel-N) fragment as the portion most likely to be involved in assembly and to precipitate at neutral pH, and the C-terminal Amel-C as a soluble domain capable of oligomerization at neutral pH.

Analytical ultracentrifugation was performed to verify self-assembly and aggregation properties of amelogenin fragments and full-length amelogenin. Our study revealed that more than 90% of the full length recombinant mouse amelogenin protein formed supramolecular assemblies at pH 7 (Fig. 7C). Among the three fragments tested in the present study, more than 50% of Amel-N self-assembled at pH 7, while the other two fragments, Amel-M and Amel-C, remained mostly monomeric (Figs. 7D–F). In contrast, at pH 5.5, all three fragments remained mostly monomeric (Figs. 7H–J), as did the full-length M180 (Fig. 7G). Our study also indicated that the assembly of the C-terminal amelogenin fragment Amel-C was very sensitive to temperature. At 25°C, the majority of Amel-C was monomeric with a small portion of tetramers (41 kDa, 7.5%), hexamers (around 70 kDa, 3.5–4.0%) and larger aggregations (146 kDa, 2%), while at 30°C, Amel-C formed very large Mega-Dalton assemblies and sedimented within several minutes of centrifugation even at low protein concentration (0.1 mg/ml) (not shown).

## Discussion

In the present study we assigned chemical shift parameters and developed a solution NMR 3D model of the major tooth enamel protein amelogenin in non-denaturing conditions. Our approach was based on obtaining NMR spectra of individual amelogenin fragments which circumvented the difficulties associated with full-length amelogenin self-assembly. Furthermore this method in tandem with deuterium enrichment enhances reliability of NOE assignments and thus provides a clear advantage for local structure determination. Moreover, chemical shift comparisons between monomeric Amel-M amelogenin and the fully assembled M180 amelogenin allowed us to identify amelogenin portions that maintained monomeric configurations in fully assembled nanospheres under physiological conditions. Our analysis identified an N-terminal assembly of four α-helical segments (M1-P60), which were not identified in previous studies, followed by an extended, random coil structure disrupted by a 3_10_ helix (P60-Q117), an elongated polyproline II helical region (P118-L165), and a hydrophilic domain (L165-D180). Further HSQC studies revealed that both the amelogenin N- and C-terminus contributed to amelogenin nanosphere structural assembly through ipsilateral interactions. Together, our studies established the separation of proteins into soluble fragments as a useful strategy to determine the solution-NMR structure of proline-rich proteins involved in self-assembly.

Cα and Hα chemical shifts value and ^3^J_HNα_-coupling values suggested the presence of α-helical structures in the amelogenin N-terminal TRAP domain (M1-W45). In addition, hNOE analysis confirmed the presence of relatively rigid configurations in this region. Others have reported similar ^3^J_HNα_-coupling values (<6 Hz), but have assigned β-turn or loop structures to the Y37-W45 segment [17]. Low J-coupling values support both α-helical and β-turn configurations; however, consistent upfield Cα and downfield Hα chemical shifts as reported in our study support the concept of α-helical conformation at the amelogenin N-terminus (M1-W45). Differences between the irregular Cα and Hα chemical shifts reported by Delak [17] and our findings may be explained by the significant differences in pH between the protein solutions used for NMR structure analysis, i.e. pH 3.8[17] compared to physiological pH (5.5) in our study. Here we suggest that the presence of N-terminal α-helices under physiological conditions is significant for amelogenin function in the developing enamel matrix. For example, the important amelogenesis imperfecta mutation P41T [27] coincides with the beginning of the third N-terminal alpha-helix. Mutation of this amino acid might thus impair the N-termial helical structure and consequentially disrupt enamel formation.

Our chemical shift assignments, J-coupling, NOE patterns, and NOESY spectra of the Amel-M molecule under near physiological conditions resolved an extended structure at the C-terminus of Amel-M. The presence of an extended structure in the amelogenin central region has been proposed in an earlier study by Delak [17] at pH 3.8. The absence of any long-range NOEs from L46 to Q154 in our NMR spectrum confirms that this region adopts an extended structure without any helices or sheets. Moreover, identical L46-M138 amelogenin HSQC spectra between the full-length and Amel-M amelogenin indicate that this region is not involved in nanosphere assembly. These results suggest that the central half of the amelogenin molecule does not directly contribute to the assembled structures and might serve other functions during enamel matrix dynamics. Using analytical ultracentrifugation, we demonstrated that rM180 formed 40 mer supramolecular assemblies (nanospheres) while Amel-M did not self-assemble; and only fractions of Amel-N and Amel-C assembled in aqueous solution.

As to the size of the supramolecular assemblies of the full-length amelogenin investigated in our studies, our calculations indicate 1046.4 kDa/26.1 kDa, i.e. 40 molecules for the full length amelogenin peak resolved by analytical ultracentrifugation. Our finding of 50 molecules per assembled subunit (40 molecules per 1064 kDa peak plus an additional 10 molecules per 1240 kDa peak) is close to a previous estimate of 100 molecules per subunit based on nanosphere diameters in AFM and TEM micrographs [9]. Explaining the discrepancy between molecule numbers per subunit, we suggest within nanosphere supramolecular assemblies, amelogenins are not densely packed, but rather form loose assemblies, facilitated by the central monomeric region (T63-M138). Our analytical ultracentrifugation studies also indicated that amelogenin N-terminal and C-terminal fragments displayed only marginal degrees of self-assembly, especially when compared to the full length amelogenin. This result was somewhat in contrast with our NMR data, which document that both the N-terminal and C-terminal ends form self-assemblies. We explain this discrepancy by proposing a fairly loose association between Amel-N or Amel-C domains, respectively. Such loose association would interfere with NMR spectra, but not persist during ultracentrifugation.

**Figure 8:**
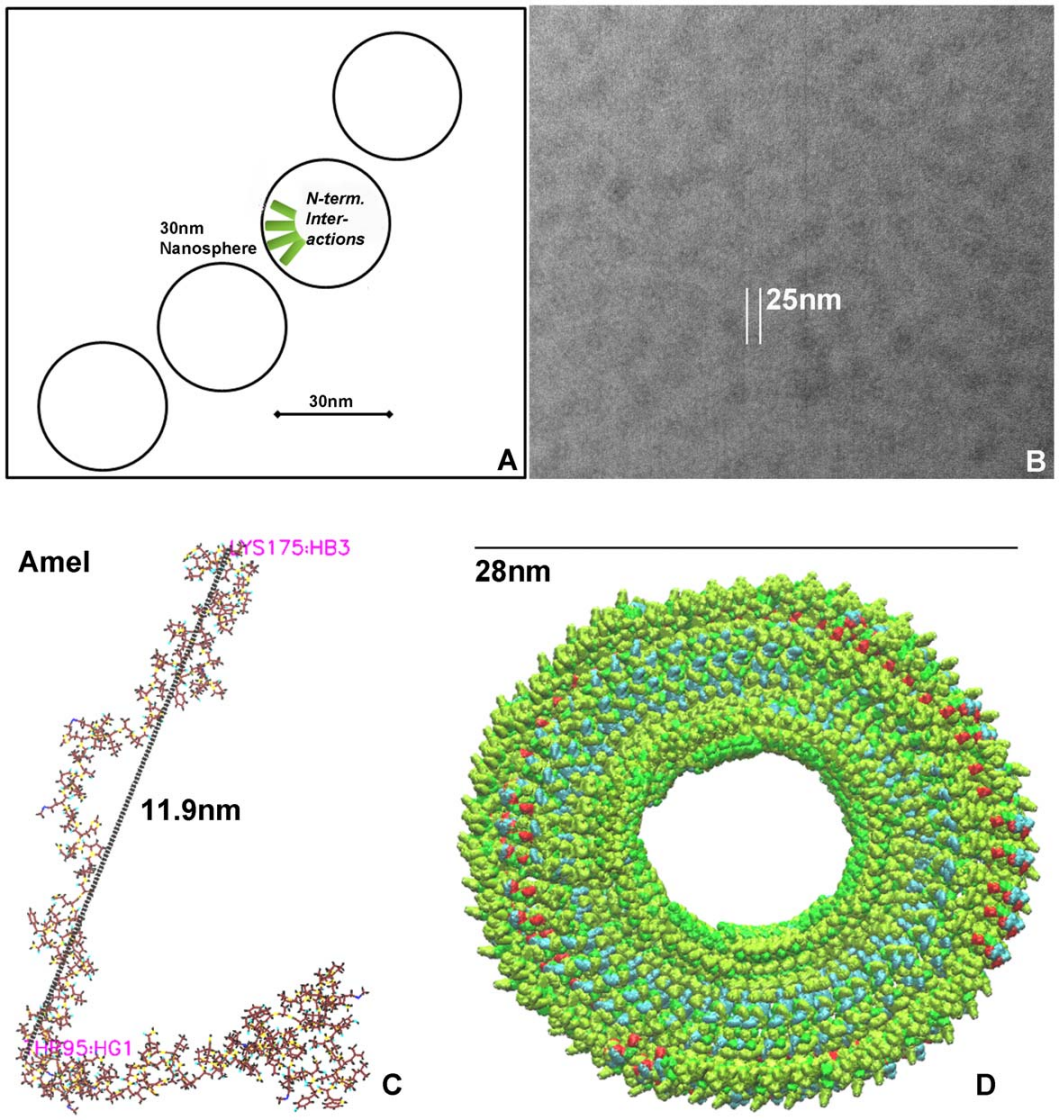
Model of basic amelogenin assembly. Chemical shift analyses in conjunction with ultracentrifugation results from this study identify the the amelogenin N-terminus as the major nanosphere assembly site (**A**). In addition, our AFM and TEM data reveal effective nanosphere diameters as 25–30 nm (**B**). Based on the analytical ultracentrifugation data presented here, we have calculated a total of 50 molecules per nanosphere (1050 kDa, major peak) and assumed 11.9 nm as the length of the full-length amelogenin length based on our NMR constraint structure (**C**). The structural model presented here (**D**) was directly generated by MolMol based on these parameters and molecules were visualized using the VMD software based on N-terminal ipsilateral interactions and weak C-terminal ipsilateral interactions. Assemblies were arranged as spheres around a hollow center region with C-termini pointing toward the outside of the assembly.

In order to visualize a basic amelogenin assembly in 3D, 50 amelogenin molecules were assembled using MolMol and visualized using the VMD software based on N-terminal ipsilateral interactions and weak C-terminal ipsilateral interactions. This model is supported by other studies suggesting N-terminal interactions as a first step in nanosphere assembly [12]. In our reconstruction, assemblies were arranged as spheres around a hollow center region with C-termini pointing toward the outside of the assembly and uplifted N-termini giving the entire assembly a collar-shaped appearance (Fig. 8). The definitive shape of amelogenin nanospheres to some degree depends on physiological conditions and methods employed. Recent studies based on SAXS and dynamic light scattering have revealed oblate shapes [28], which might form functionally important intermediaries between spherical and sheeded assemblies. Our concept of hollow nanospheres is in congruence with recent cryo-TEM data [29].

Together, our 3D NMR studies suggest that amelogenin nanospheres are oligomolecular assemblies facilitated by strong interactions between N-terminal helices. Interference spectroscopy with opposite domains document that amelogenin molecules favor ipsilateral interactions on both ends of the molecule. The presence of intermolecular interactions at the C-terminus as a second interaction domain suggests that nanosphere-assemblies are stabilized by interacting C-termini, likely toward the outside of the assembly. In this model, the center of the molecule might function as a hinge during molecular interactions involved in calcium phosphate assembly. Within individual assemblies, the long PXX repeat region may serve as an expansion domain, defining the size of the nanosphere as previously described [9], while the central region from T63 to M138 is not involved in any interaction during nanosphere assembly.

## Materials and Methods

### Materials

Expression vectors pASK-43(+) was purchased from The TAG company (Göttingen,Germany). The ^13^C labeled D-GLUCOSE, ^15^N labeled amide chloride and D_2_O (99.5%) was purchased from Cambridge Isotope Laboratories (Andover, MA). 400 MHz PCR tubes were obtained from Kontes (Vineland, NJ). Other common regents were from Sigma Aldrich (St Louis, MO).

### Cloning and expression of mouse full length amelogenin and its three overlapping fragments

The full length mouse amelogenin and its three overlapping fragments were cloned into pASK-43(+). The expressed proteins have a HIS-tag of MRGSHHHHHHGAGDRGPE at the N-terminus of the protein. BL21-DM* was used as the host bacteria to express the recombinant proteins. The bacteria were cultured at 37°C until the OD_600_ reached 0.8 and then were induced at 32°C for 3∼4 hours. The ^15^N, ^13^C, ^2^H incorporated proteins were expressed in M9 basic media with ^15^N labeled amide chloride, ^13^C labeled D-glucose as the nitrogen and carbon source, using D_2_O instead of H_2_O to prepare the media. The expressed proteins were absorbed onto Ni-NTA agarose column and wash with 10 column volumes of PBS and 3 column volumes of 40 mM imedazole in PBS, then the proteins were eluted using a pH gradient (pH 6.8–4.9) and imidazole-PBS solution. The eluted proteins dialyzed against H_2_O several times to make sure the salt and imidazole were diluted at least 10,000 times. Then the purified proteins were concentrated to around 10 mg/ml with Centriprep YM-3 column.

### Nuclear Magnetic Resonance

NMR measurements were performed with the presence of 10% D_2_O at pH 5.5 (5 mM NaH_2_PO_4_ and 0.02% Acetate Acid) at 25°C on a Bruker Avance 900 MHz or Bruker DRX 600 MHz spectrometer. The concentration of the proteins was about 10 mg/ml. Standard ^1^H-^15^N HSQC experiments were conducted to evaluate the most favorable NMR conditions. Various 2D and 3D experiments including HSQC-TOCSY, HSQC-NOESY, HNCA, CBCACONH, HN(CA)CB, HBHA, HNCO, h-TOCSY, HN(CO)CA C-TOCSY were performed in order to generate backbone, side chain and NOE constraint assignments. The mixing times for TOCSY and NOESY were 80 ms and for NOESY-HSQC 120 ms and 150 ms respectively. The HNHA experiment was used to measure ^3^J_HNα_-coupling values. Both saturated and unsaturated HSQC (heteronuclear NOE) experiments were conducted to evaluate the backbone flexibility of each amino acid residue. In addition, iPAP experiments were performed with PEG as the semicrystal carrier. Spectra were processed and analyzed using the SYBYL software package (Tripos, MO) or NMRPipe. All ^1^H and ^13^C dimensions were referenced to internal 2,2-dimethyl-2-silapentane-5-sulfinate (DSS). NOE constraints were manually classified into strong (2Å), medium (4Å), and weak (6Å) groups. Peaks generated from obvious spin diffusion in NOESY-HSQC (150 ms) were excluded. Structure calculations were performed with the DYANA 1.5 program [30], using a 40,000-step energy minimization procedure. The obtained structures were further refined with NMR constrain calculations using SANDER from the AMBER software package.

### Molecular dynamics (MD) calculation

The 10 structures with the lowest energy obtained by DYANA simulation were further refined by NMR constrain-based molecular dynamics calculations using SANDER from the AMBER software package [31]. The Generalized Born (GB) solvation model was applied to the molecular dynamics simulations to reduce the computational time. The GB solvation model provides an approximate solution to the solute-solvent electrostatic polarization term without costly computations of numerical solutions. Total 2 ns time window was calculated and structures were refined with 970 NOE distance constraints and 68 angle (PHI and PSI) constraints. All subsequent analyses of the structure and graphic representations of the three-dimensional structures were performed using MOLMOL [32] and VMD [33].

### Analytical ultracentrifugation

Sedimentation Velocity experiments were carried out using an Optima XL-A analytical ultracentrifuge (Beckman Coulter, Fullerton, CA) equipped with a Ti60 rotor. SV data for amelogenins and fragments were obtained at 40,000 rpm, 25°C, using an Epon two-channel centerpiece. Absorbance of the samples at 280 nm was monitored in a continuous mode time interval of 360–480 s and a step size of 0.003 cm. Multiple scans at different time points were fitted to the continuous size distribution (c(s)) model using SEDFIT 11.3. The partial specific volume of the proteins and buffer density were calculated using SEDNTERP. Molecular mass determination was performed at 2 mg/ml in 10 mM Tris buffer for Amel-N, Amel-M, Amel-C and full length recombinant mouse amelogenin at both ∼pH 7.0 and pH 5.5.

### 3D model of nanosphere

Our mouse amelogenin self-assembly nanosphare model was established using the MOLMOL software and viewed with the VMD software. The following four parameters were entered into our MolMol structure calculation: (i) 50 molecules per nanosphere based on our analytical ultracentrifugation data, (ii) 20–30 nm diameter nanospheres based on TEM and AFM data (TEM ∼20 nm and AFM ∼30 nm; suggesting 30 nm because of the hydrated state of nanospheres [9], (iii) full-length amelogenin length as 11.9 nm based on our NMR constraint structure, and (iv) N-terminal assembly based on the NMR data presented in this paper. The structural model presented here (Fig. 8D) was directly generated by MolMol based on these parameters.

## Supporting Information

Figure S1 **Graphic illustration of amelogenin chemical shifts.** Plots illustrate the chemical shift deviations from experimental chemical shifts and predicted chemical shifts (ppm). ΔδHN  =  HN (experiment chemical shifts) – HN (predicted chemical shifts); ΔδCα = Cα (experiment chemical shifts) – Cα (predicted chemical shifts); ΔδN = N (experiment chemical shifts) – N (predicted chemical shifts); ξ = ((ΔδHN2+ δCα2/4+ΔδN2/25)/3)0.5.
(PDF)

Table S1 **Chemical shift comparisons between overlapping areas of the three amelogenin fragments employed in the present study.** This table confirms good matches of chemical shift measurements between his-tagged flanking regions of fragments and mid-fragment coordinates.
(PDF)
