# Supplementary figures and images for: Amelogenin Supramolecular Assembly in Nanospheres Defined by a Complex Helix-Coil-PPII Helix 3D-Structure
Source: PLoS One. 2011 Oct 3;6(10):e24952. doi: 10.1371/journal.pone.0024952 (PMC3184955; doi:10.1371/journal.pone.0024952)

**Figure S1. Graphic Illustration of Amelogenin Chemical Shifts.**

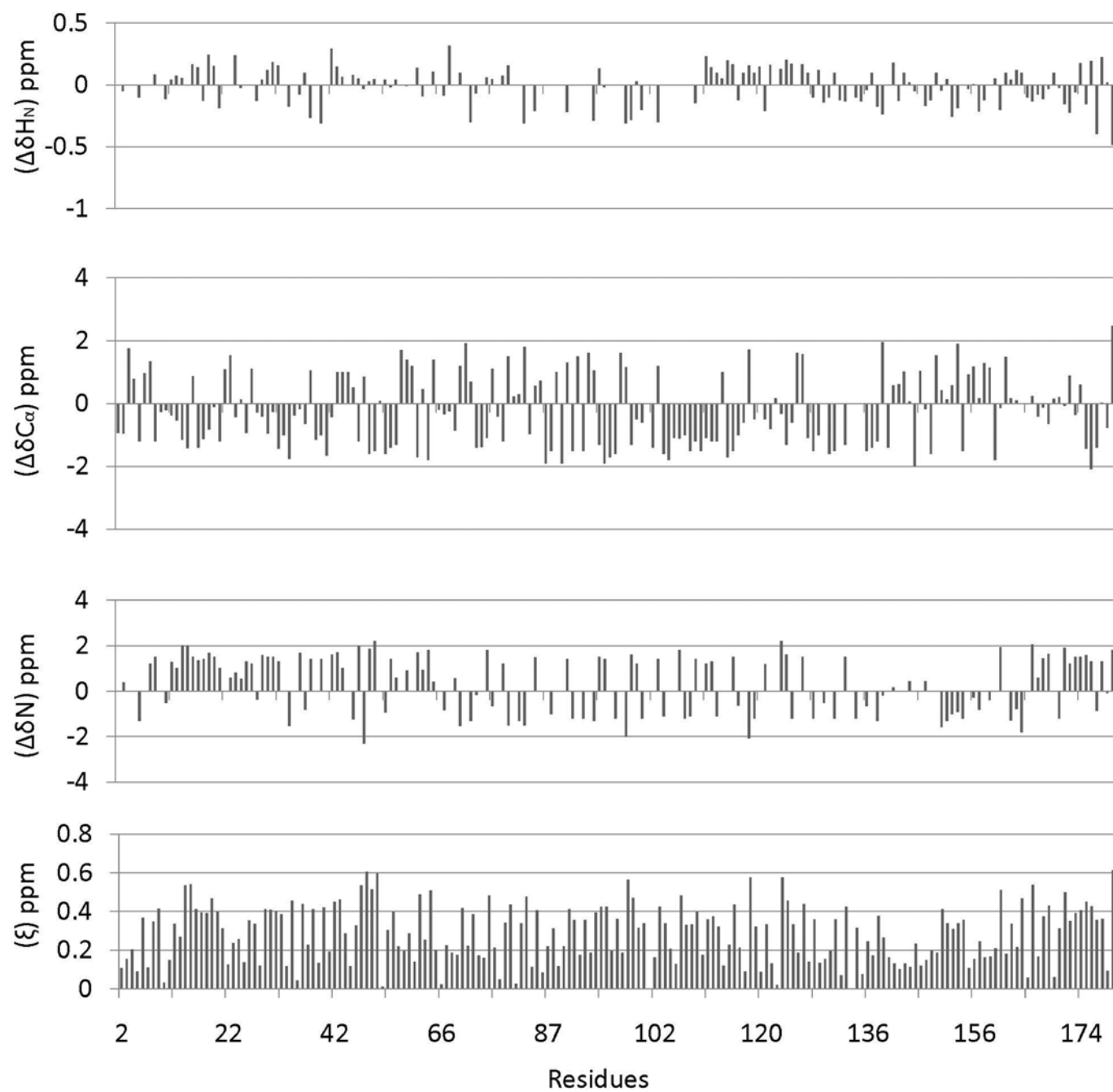

Supplement: Figure S1 — Graphic illustration of amelogenin chemical shifts. Plots illustrate the chemical shift deviations from experimental chemical shifts and predicted chemical shifts (ppm). ΔδHN = HN (experiment chemical shifts) – HN (predicted chemical shifts); ΔδCα = Cα (experiment chemical shifts) – Cα (predicted chemical shifts); ΔδN = N (experiment chemical shifts) – N (predicted chemical shifts); ξ = ((ΔδHN2+ δCα2/4+ΔδN2/25)/3)0.5. (PDF) [file pone.0024952.s001.pdf]
