# Supplementary material for: Amelogenin Supramolecular Assembly in Nanospheres Defined by a Complex Helix-Coil-PPII Helix 3D-Structure
Source: PLoS One. 2011 Oct 3;6(10):e24952. doi: 10.1371/journal.pone.0024952 (PMC3184955; doi:10.1371/journal.pone.0024952)
Supplement: Table S1 — Chemical shift comparisons between overlapping areas of the three amelogenin fragments employed in the present study. This table confirms good matches of chemical shift measurements between his-tagged flanking regions of fragments and mid-fragment coordinates. (PDF) [file pone.0024952.s002.pdf]

Table S1. Comparison between chemical shifts between overlapping areas of the three amelogenin fragments employed in the present study

HSQC	N-domain	M-domain	C-domain
**Y34**	H7.8 ppm N122 ppm	H7.8 ppm N123 ppm	
**Y37**	H7.97 ppm N121.2 ppm	H7.97 ppm N121.3 ppm	
**Y39**	H7.8 ppm N119.6 ppm	H7.8 ppm N119.8 ppm	
**H91**		H8.2 ppm N118.2 ppm	H8.1 ppm N119 ppm
**T95**		H8.17 ppm N118.2 ppm	H8.17 ppm N118.2 ppm
**T97**		H8.1 ppm N114.4 ppm	H8.1 ppm N114.2 ppm
